# Intravenous Injection of PHF-Tau Proteins From Alzheimer Brain Exacerbates Neuroinflammation, Amyloid Beta, and Tau Pathologies in 5XFAD Transgenic Mice

**DOI:** 10.3389/fnmol.2020.00106

**Published:** 2020-07-14

**Authors:** Sarah Houben, Marie-Ange de Fisenne, Kunie Ando, Virginie Vanden Dries, Luc Poncelet, Zehra Yilmaz, Salwa Mansour, Robert De Decker, Jean-Pierre Brion, Karelle Leroy

**Affiliations:** ^1^Laboratory of Histology, Neuroanatomy and Neuropathology, Faculty of Medicine, ULB Neuroscience Institute, Université Libre de Bruxelles, Brussels, Belgium; ^2^Laboratory of Anatomy, Biomechanics and Organogenesis, Faculty of Medicine, ULB Neuroscience Institute, Université Libre de Bruxelles, Brussels, Belgium

**Keywords:** tau, neurofibrillary tangles, amyloid, plaque, intravenous, blood transfusion

## Abstract

Alzheimer’s disease (AD) is characterized by the accumulation in the brain of intraneuronal aggregates of abnormally and hyperphosphorylated tau proteins and of extracellular deposits of amyloid-β surrounded by dystrophic neurites. Numerous experimental models have shown that tau pathology develops in the brain after intracerebral injection of brain homogenates or pathological tau [paired helical filaments (PHF)-tau)] from AD brains. Further investigations are however necessary to identify or exclude potential extracerebral routes of tau pathology transmission, e.g., through the intravascular route. In this study, we have analyzed the effect of intravenous injection of PHF-tau proteins from AD brains on the formation of tau and amyloid pathologies in the brain of wild-type (WT) mice and of 5XFAD mice (an amyloid model). We observed that 5XFAD mice with a disrupted blood–brain barrier showed increased plaque-associated astrogliosis, microgliosis, and increased deposits of Aβ40 and Aβ42 after intravenous injection of PHF-tau proteins. In addition, an increased phosphotau immunoreactivity was observed in plaque-associated dystrophic neurites. These results suggest that blood products contaminated by PHF-tau proteins could potentially induce an exacerbation of neuroinflammation and AD pathologies.

## Introduction

Alzheimer’s disease (AD) is a neurodegenerative disease characterized by two neuropathological lesions called neurofibrillary tangles (NFT) and senile plaques. NFT are composed of abnormally and hyperphosphorylated tau proteins that form aggregates in neurons as paired helical filaments (PHF; Brion et al., 1985; Grundke-Iqbal et al., 1986; Buée et al., 2000). The cognitive deficits are highly correlated to the abundance of NFT in the brain of AD subjects (Nelson et al., 2012). Senile plaques are composed of extracellular deposits of the amyloid-β peptide (Aβ) surrounded by dystrophic neurites containing abnormally phosphorylated tau proteins. Aβ is produced by the proteolytic cleavage of the amyloid precursor protein (APP; Glenner and Wong, 1984; Kang et al., 1987).

Experimental models have demonstrated that tau pathology propagates in the brain after exposure to pathological tau in a prion-like manner by recruiting endogenous tau (Mudher et al., 2017). Indeed, the seeding of endogenous murine or human tau has been observed in the brain after intracerebral injection of brain homogenates or PHF-tau proteins from AD brain tissues in wild-type (WT) and in tau transgenic mice (Clavaguera et al., 2013, 2014; Audouard et al., 2016).

Further investigations are necessary to identify potential extracerebral routes of tau pathology transmission as it was suggested for some prion diseases. Indeed, cases of iatrogenic Creutzfeldt–Jakob’s disease (CJD) have been induced by the use of contaminated growth hormone derived from cadaver with undiagnosed CJD infections and by transfusion of blood products (Brown, 1988; Llewelyn et al., 2004; Wroe et al., 2006; Brown et al., 2012). In some cases of iatrogenic CJD, tau pathology was observed in the brain of patients after exposure to cadaver-derived human growth hormone, suggesting that tau pathology could develop through an extracerebral iatrogenic transmission (Duyckaerts et al., 2018). The role of the intravascular system as a potential extracerebral route for transmission of tau pathology is not clearly elucidated as studies showed no association or an increased risk for AD after blood transfusion in cohort studies (O’Meara et al., 1997; Edgren et al., 2016; Lin et al., 2019). The presence of phosphorylated tau proteins has however been demonstrated in the plasma of normal subjects (Yang et al., 2018). Plasma tau protein was detected already in young individuals and the concentration of plasma tau proteins increased with age in cognitively normal subjects (Chiu et al., 2017; Lue et al., 2019). Moreover, the permeability of the blood–brain barrier has been reported to be increased with age in the hippocampus of healthy individuals (Montagne et al., 2015) and in AD (Rosenberg, 2014; van de Haar et al., 2016), suggesting that aged individuals with compromised blood–brain barrier might be more susceptible to passage of plasma tau proteins in the brain and potential seeding of a tau pathology. The presence of an amyloid-β pathology in the brain might also facilitate the seeding of a tau pathology, since recent studies have shown that amyloid increases tau seeding after intracerebral injection of PHF-tau proteins from AD brain (He et al., 2018; Vergara et al., 2019). To investigate experimentally a potential role of the intravascular route in the development of a tau pathology in a model with compromised blood–brain barrier and amyloid-β pathology, we have analyzed amyloid and tau pathologies after intravenous injection of PHF-tau proteins from AD brain in WT and 5XFAD mice. 5XFAD mice, an animal model of AD amyloidosis expressing human mutated APP and Presenilin 1 (PS1), develops amyloid plaques in the brain at 3 months of age (Oakley et al., 2006) and show a higher permeability of the blood–brain barrier (Kook et al., 2012, 2013; Batarseh et al., 2017). This higher permeability in 5XFAD mice is due to the presence of Aβ42, which induces a disruption of tight junctions in brain capillaries and has also been observed in AD brains (Wisniewski et al., 1997; Kook et al., 2012, 2013; Batarseh et al., 2017; Yamazaki et al., 2019). We observed that a single intravascular injection of PHF-tau proteins induced a long-term inflammatory response in 5XFAD mice, an increased amyloid-β burden and increased tau immunoreactivity in plaque-associated dystrophic neurites.

## Materials and Methods

### Human Brain Tissue

Human brain tissue samples were taken at autopsy from a demented patient clinically diagnosed as having AD (60 years old, female, postmortem delay of 24 h) or from a nondemented control subject (67 years old, male, postmortem delay of 24 h). Tissue samples were fixed with formalin 10% and embedded in paraffin for neuropathological examination or were kept at −80°C. The neuropathological examination confirmed the presence of NFT and amyloid plaques in this AD case (Braak stage VI, Thal stage 4) and their absence in the control case. This study on postmortem brain tissue was performed in compliance and following approval of the Ethical Committee of the Medical School of the Free University of Brussels.

### Preparation of Human Sarkosyl-Insoluble PHF-Tau Fraction

Sarkosyl fractionation of human brain tissue was performed as previously described (Brion et al., 1991; Frederick et al., 2015). Frozen frontal cortex (0.5 g) from AD and control cases was homogenized in 10 volumes of ice-cold PHF-extraction buffer [10 mM Tris–HCl (pH 7.4), 0.8 M NaCl, 1 mM EDTA, and 10% sucrose]. The homogenate was centrifuged at 15,000× *g* for 20 min at 4°C. N-lauroylsarcosine sodium salt (L-5125; Sigma-Aldrich) was added to the supernatant to reach a final concentration of 1% (w/v). The lysate was incubated overnight at 4°C with a mild agitation followed by an ultracentrifugation at 180,000× *g* for 30 min at 4°C. The sarkosyl soluble supernatant was removed and the sarkosyl-insoluble pellet, containing PHF, was gently rinsed and re-suspended in 0.25 ml of PBS by vigorous pipetting. The protein concentration was determined by Bradford protein assay (Bio-Rad). These Sarkosyl fractions were aliquoted and kept at −20°C.

### Negative Staining of Tau Filaments by Transmission Electron Microscopy

The Sarkosyl-insoluble material was ultrastructurally characterized by transmission electron microscopy. This material was adsorbed on formvar-carbon-coated EM grids and negatively stained with potassium phosphotungstate as reported before (Brion et al., 1991; Poncelet et al., 2019) and observed with a Zeiss EM 809T at 80 kV. The average length of sarkosyl-insoluble filaments was measured on 200 filaments, using the ImageJ software.

### Animals

The 5XFAD heterozygote mice contain five familial AD mutations for APP (K670N/M671L, I716V, V717I) and for PS1 (M146L, L286V; Oakley et al., 2006). Mutants APP and PS1 transgene expression is driven by the mouse Thy1 promoter. Genotyping was performed by PCR amplifications of DNA extracted from tail, using previously described primers for human APP (Oakley et al., 2006; Leroy et al., 2012). Only female heterozygote animals were used in the present study; non-transgenic littermates were used as WT controls. Tg30 mice express 1N4R human tau mutated on G272V/P301S under the control of a Thy.1 promoter (Schindowski et al., 2006; Leroy et al., 2007). Brain sections of these mice were used as positive control for anti-human or pathological tau immunolabelings.

### Intravenous Injection of Sarkosyl Fractions

Three-month-old WT and 5XFAD female mice were not treated (not injected group: WT mice, *n* = 3; 5XFAD mice, *n* = 3) or treated by injection in the orbital venous plexus of 10 μg proteins of sarkosyl fraction isolated from control frontal cortex (CTL injected group: WT mice, *n* = 3; 5XFAD mice, *n* = 3) or sarkosyl fraction isolated from AD frontal cortex (AD injected group: WT mice, *n* = 3; 5XFAD mice, *n* = 3). Six months after injection, mice were anesthetized with a solution of xylazine (5% v/v; Rompun, Bayer) and ketamine hydrochloride (10% v/v; Nimatek) in physiological saline by i.p. injection (100 ml/10 g of body weight, final dose, 10 mg/kg xylazine, and 100 mg/kg ketamine) and the blood was retrieved by intracardiac punction and allowed to coagulate. Tubes containing coagulated blood was centrifuged at 1000× *g* for 10 min at room temperature. The supernatant corresponding to serum was retrieved. Brains were fixed in 10% formaldehyde and embedded in paraffin. All studies on animals were performed in compliance and following approval of the Ethical committee for the care and use of laboratory animals of the Medical School of the Free University of Brussels.

### Western Blotting

The protein assay was performed with the Bradford method (Biorad). Sarkosyl-insoluble fractions from control or AD frontal cortex were heated in Laemmli buffer at 100°C for 5 min and were analyzed by Western blotting, with anti-total tau B19, anti-phosphotau PHF-1, and anti-amyloid antibodies. Fractions (5 μg/lane) were run in 10% Tris–glycine SDS-PAGE gels and transferred on nitrocellulose membranes (Leroy et al., 2000). The nitrocellulose sheets were blocked in semifat dry milk [10% (w/v) in Tris-buffered saline] for 1 h at room temperature and incubated overnight with primary antibodies, followed by anti-rabbit or anti-mouse immunoglobulins conjugated to peroxidase. Finally, the membranes were incubated in pico substrate (Pierce). The ECL signal was captured using a Fusion SOLO 4S system equipped with a DARQ-7 camera and the fusion-capt software (Vilber-Lourmat).

### Antibodies and Immunohistochemistry

The characteristics of all primary antibodies used in this study are summarized in Supplementary Table S1.

Tissue sections (7 μm of thickness) were deparaffined and incubated in H_2_O_2_ to inhibit endogenous peroxidase. Sections were rinsed in water and then incubated with a blocking solution [10% normal rabbit serum in TBS (Tris 0.01 M, NaCl 0.15 M, pH 7.4)]. After this incubation, sections were incubated overnight with the primary antibody solution and then incubated with anti-mouse or anti-rabbit antibodies conjugated to biotin (Vector) followed by the ABC complex (Vector). Sections were revealed with a peroxidase substrate, a solution of diaminobenzidine as a chromogen (DAKO). Sections immunolabeled with anti-amyloid antibodies were pre-treated with 99% formic acid (Stygelbout et al., 2014). Tissue sections were stained with hematoxylin, Thioflavin T, Gallyas staining, or DAPI for histological examination as previously described (Ando et al., 2011; Leroy et al., 2012; Poncelet et al., 2019).

For detection of immunoglobulin extravasation, sections were incubated directly with anti-mouse antibody conjugated to biotin (Vector) followed by the ABC complex (Vector) without primary antibody. Sections were revealed with a peroxidase substrate, a solution of diaminobenzidine as a chromogen.

### Quantification of Labeling

Mouse brain sections were labeled with the corresponding antibody or stained and examined with an Axioplan microscope (Carl Zeiss). Digital images covering all the dentate gyrus of the hippocampus were acquired with a 2.5× objective lens, an Axiocam Hrc camera, and the Axiovision software (Carl Zeiss). The total surface of the dentate gyrus was measured using manual selection and the labeled area in the dentate gyrus was determined with the ImageJ software (NIH) by detecting positive pixels using image thresholding, as reported previously (Héraud et al., 2014; Vanden Dries et al., 2017; Houben et al., 2019).

### Statistical Analysis

Statistical analyses were performed using Prism 7 software (GraphPad Software). Statistical comparisons were performed using one-way or two-way ANOVA followed by Tukey’s Multiple Comparison Test as noted in figure legends. Values of *p* < 0.05 were considered significant.

## Results

### Characterization of Sarkosyl-Insoluble Fraction From Human Brain

**Figure 1:**
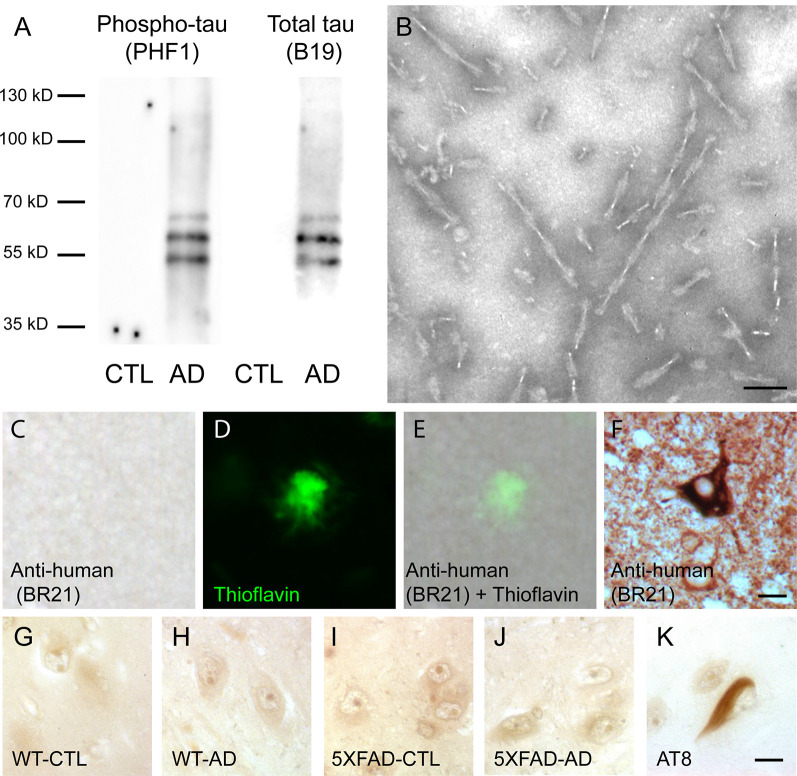
Intravenous injection of paired helical filaments (PHF)-tau proteins does not induce anti-tau immunoglobulins production. **(A)** Western blotting of sarkosyl-insoluble fraction from control (CTL) or Alzheimer’s disease (AD) brain containing PHF-tau with antiphosphotau antibody (PHF1) or total tau antibody (B19). Tau immunoreactivity was detected in sarkosyl fraction from AD brain but not in sarkosyl fraction from CTL brain. **(B)** Sarkosyl-insoluble fraction from AD brain contains PHF observed by transmission electron microscopy. Scale bar: 75 nm. **(C–F)** Immunolabeling on tissue sections of the hippocampus of 5XFAD mice injected with AD sarkosyl fractions with anti-human tau antibody **(C,E)** and counterstained with thioflavin to stain amyloid plaques **(D,E)**. Tg30 mice brain sections expressing human mutant 1N4R tau were used as a positive control for anti-human tau immunolabeling **(F)**. Scale bar: 10 μm. **(G–K)** Immunolabeling on tissue sections of the hippocampus of an AD patient with the serum of wild-type (WT; **G,H**) or 5XFAD mice **(I,J)** injected with sarkosyl fractions from control **(G,I)** or AD **(H,J)** brain. Serums from injected mice do not show any tau immunoreactivity on the hippocampus of this AD patient. AT8 immunolabeling was used as a positive control **(K)**. Scale bar: 10 μm.

The presence of tau in sarkosyl fractions from control or AD brains that were used for injection was analyzed by Western blotting with anti-phosphotau (PHF1) and anti-total tau antibodies (Figure 1A). We detected phosphorylated tau proteins (PHF-tau proteins) as three major bands as previously described in AD sarkosyl fractions (Goedert et al., 1992). These bands were absent in sarkosyl fractions from control subjects. The presence of tau filaments in the form of PHF in sarkosyl fractions from AD brain was confirmed by negative staining by electron microscopy (Figure 1B). The PHF had an average length of 165.9 ± 7.6 nm (mean ± SEM). We also analyzed the sarkosyl fractions for the presence of amyloid-β by Western blotting with the 6E10 antibody. Amyloid-β was not detected in sarkosyl fractions (data not shown).

### Single Intravenous Injection of PHF-Tau Proteins Does Not Induce the Production of Anti-tau Antibodies in WT and 5XFAD Injected Mice

In order to test for the presence of anti-tau antibodies in the serum of intravenously injected mice, we labeled brain sections of AD brains with serums of control or AD injected mice using them as primary antibodies. Tau immunoreactivity was not detected on AD brain sections labeled with serum of WT or 5XFAD injected mice (Figures 1G–J), whereas NFT were detected with AT8 anti-phosphotau antibody on an adjacent section of AD brain (Figure 1K).

### Intravenous Injection of PHF-Tau Proteins Increases Astrocytic and Microglial Immunoreactivity in 5XFAD Mice Brain

**Figure 2:**
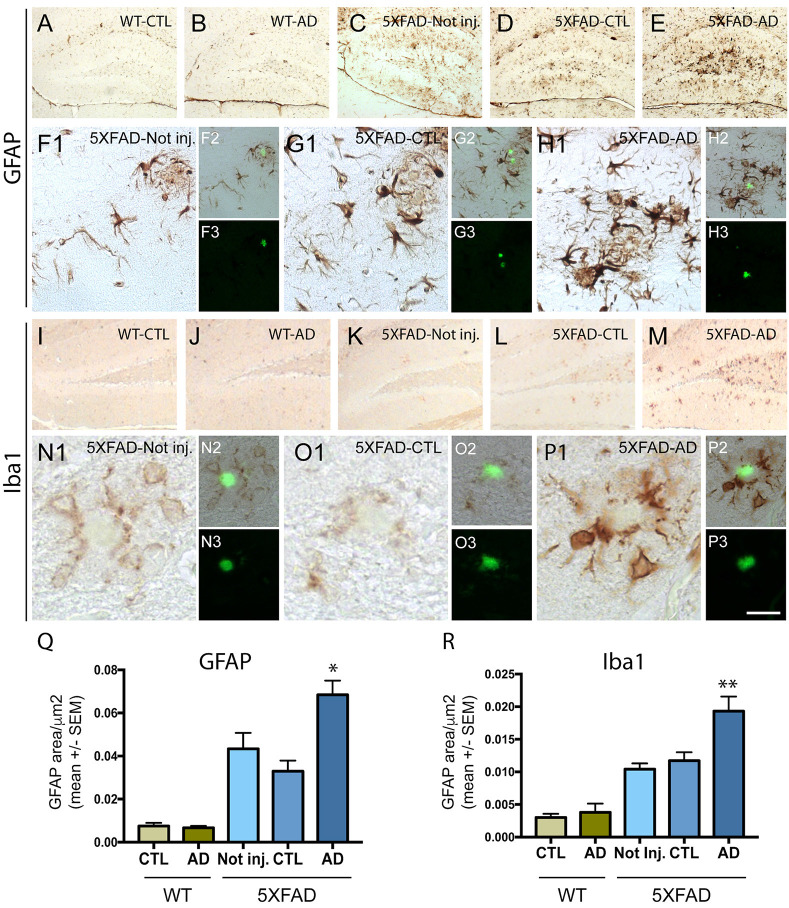
Neuroinflammation is increased in 5XFAD mice after injection of PHF-tau proteins from AD brain. (**A**–**H3**): Immunolabelling with anti-GFAP antibody on the dentate gyrus of WT mice **(A,B)** and 5XFAD mice (**C**–**H3)** not injected (**C** and **F1–3**) or injected with sarkosyl fractions from control (**A**,**D** and **G1–3**) or AD brain (**B,E** and **H1–3**). Pictures (**F1**,**G1** and **H1**) are higher magnifications of the dentate gyrus illustrated in (**C**,**D** and **E**), respectively. Sections were counterstained with Thioflavin T (**F3**,**G3** and **H3**) and showed localization of astrocytic cells **(F1,G1,H1)** around amyloid deposits (in green in figures **F2,G2** and **H2**). (**I**–**P3**) Immunolabelling with anti-Iba1 antibody on the dentate gyrus of WT mice (**I** and **J**) and 5XFAD mice (**K**–**P3**) not injected (**K** and **N1–3**) or injected with sarkosyl fractions from control (**I,L** and **O1–3**) or AD brain (**J,M** and **P1–3**). Pictures (**N1**,**O1** and **P1**) are higher magnifications of the dentate gyrus illustrated in (**K,L** and **M**), respectively. Sections were counterstained with Thioflavin T (**N3**,**O3** and **P3**) and showed localization of microglial cells around amyloid deposits (in green in figures **N2**,**O2** and **P2**). Scale bars: (**A–E**,**I–M**): 250 μm and (**F1–H1**,**N1–P1**): 40 μm, N2/3-P2/3: 80 μm. (**Q**,**R**) Quantification of GFAP **(Q)** and Iba1 **(R)** immunostainings in the dentate gyrus of WT and 5XFAD mice not injected or injected with sarkosyl fractions from control or AD brain. 5XFAD mice injected with sarkosyl fractions from AD brain showed a significant increase of the GFAP **(Q)** or the Iba1 **(R)** immunolabelling compared to control or AD injected WT mice or compared to 5XFAD mice not injected or injected with control fraction (Two way anova, Tukey post-test, *p < 0.05; **p < 0.01). No significant differences were observed in WT mice injected with sarkosyl fractions from control or AD brain or in 5XFAD mice not injected or injected with sarkosyl factions from control brain.

To understand the effect of intravenous injection of PHF-tau into WT and 5XFAD mice, we first investigated glial activation by immunohistochemistry using anti-GFAP (an astrocytic marker) and anti-iba1 (microglial or macrophage marker) antibodies (Figure 2). A prominent astrocytosis and microgliosis were observed in the dentate gyrus of 5XFAD mice injected with AD sarkosyl fractions (Figures 2E,H,M,P) and significantly increased compared to WT mice injected with control or AD sarkosyl fractions, or even compared to 5XFAD not injected or injected with control sarkosyl fraction (Figures 2Q,R). The reactive astrocytes and microglial cells were mainly localized around amyloid plaques in 5XFAD mice injected with PHF-tau proteins from AD (Figures 2H,P).

### Intravenous Injection of PHF-Tau Proteins Exacerbates Amyloid Plaque Burden

**Figure 3:**
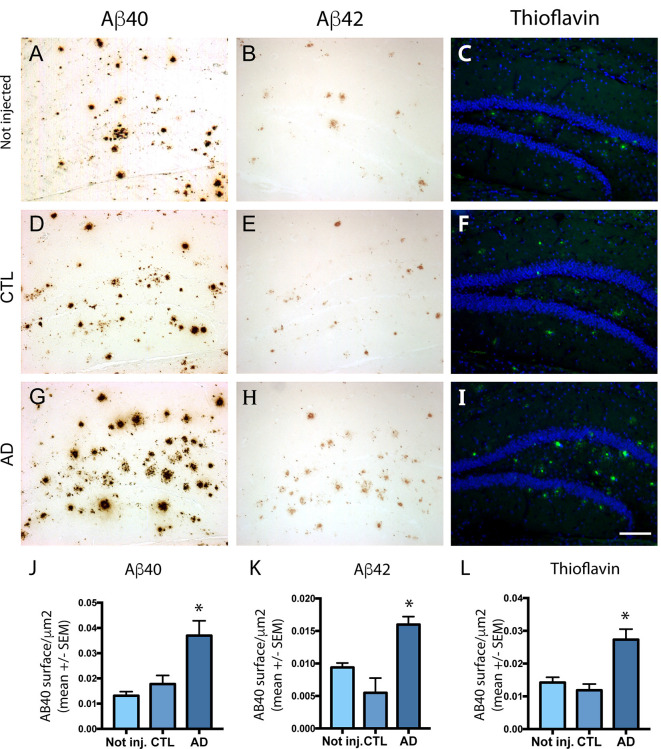
Effect of intravenous injection of PHF-tau proteins from AD brain on the formation of amyloid plaques. Immunolabeling of the dentate gyrus of 5XFAD mice not injected **(A–C)** or injected with sarkosyl fractions from control **(D–F)** and AD brain **(G–I)** with anti-Aβ40 **(A,D,G)**, anti-Aβ42 **(B,E,H)** antibodies and with Thioflavin T staining **(C,F,I)**. Quantification of Aβ40, Aβ42, and Thioflavin T immunostaining showed a significant increase in 5XFAD mice injected with sarkosyl fraction from AD brain compared to 5XFAD mice not injected or injected with control fraction (**J–L**; one-way ANOVA, Tukey post-test, *p < 0.05). Slides were counterstained with DAPI in pictures **(C,F,I)**. Scale bar: 200 μm.

To analyze the effect of intravenous injection of PHF-tau proteins on amyloid plaque formation, we quantified by immunohistochemistry the load of amyloid plaques in non-injected or injected 5XFAD mice with antibodies to Aβ40 and to Aβ42 and with thioflavin staining (Figure 3). The mean area covered by Aβ40 (Figures 3A,D,G) and Aβ42 (Figures 3B,E,H) positive plaques was significantly increased in the dentate gyrus of 5XFAD mice injected with AD sarkosyl fraction compared to 5XFAD mice not injected or injected with control fraction (Figures 3J,K). The increase of amyloid plaque burden in 5XFAD mice injected with AD sarkosyl fraction was also observed when using thioflavin T staining (Figures 3C,F,I,L).

### Effect of Intravenous Injection of PHF-Tau Proteins on the Development of Tau Pathology

**Figure 4:**
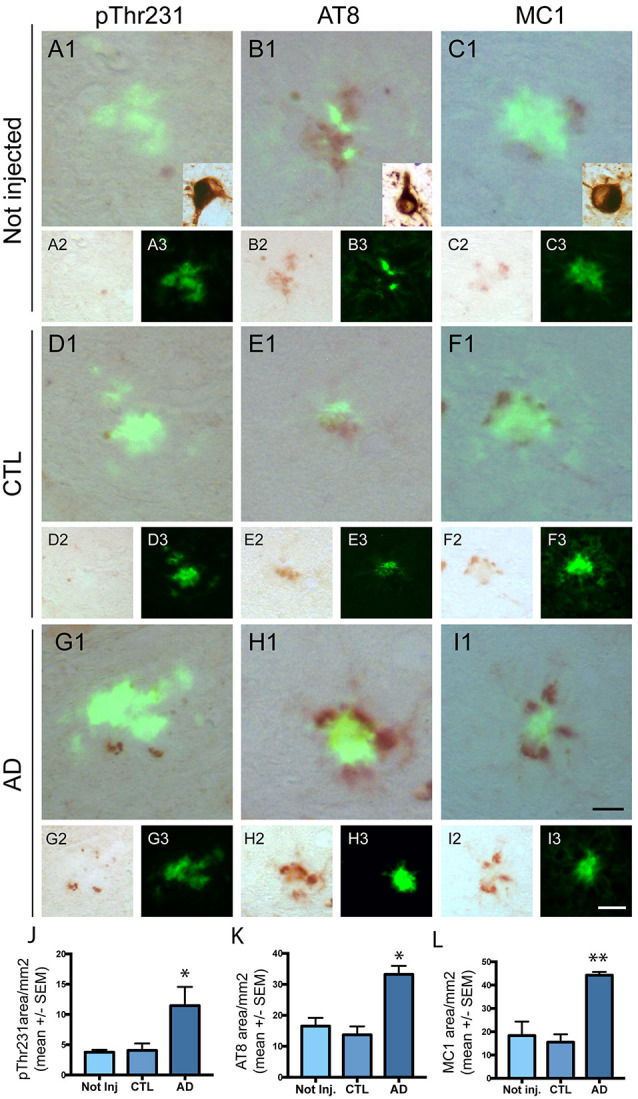
Intravenous injection of PHF-tau proteins exacerbates tau pathology in 5XFAD mice. Immunolabelling of the dentate gyrus of 5XFAD mice not injected **(A1–3,B1–3 and C1–3)** or injected with sarkosyl fractions from control **(D1–3,E1–3 and F1–3)** and AD brain **(G1–3,H1–3 and I1–3)** with anti-phosphoThr231 antibody **(A1/A2,D1/D2 and G1/G2)**, AT8 antibody **(B1/B2,E1/E2 and H1/H2)** and with MC1 antibody **(C1/C2,F1/F2 and I1/I2)**. Quantification of phospho-Thr231, AT8 and MC1 immunostaining in dystrophic neurites surrounding amyloid plaques showed a significant increase in 5XFAD mice injected with sarkosyl fraction from AD brain compared to 5XFAD mice not injected or injected with control fraction (**J,K** and **L**; One-way anova, Tukey post-test, *p < 0.05; **p < 0.01). Slides were counterstained with Thioflavin T to show amyloid deposits in green. Tg30 mice brain expressing human mutant 1N4R tau was used as a positive control for immunolabellings (insets in **A1,B1 and C1**). Scale bar: 20 μm.

We next investigated the effect of intravenous injection of PHF-tau proteins on the formation of tau pathology in the brain of injected mice. We never detected accumulation of phosphorylated tau in WT mice injected with control or AD sarkosyl fractions (data not shown). The presence of phosphorylated and conformationally modified tau was detected with pThr231, AT8, and MC1 antibodies in dystrophic neurites surrounding amyloid plaques in 5XFAD mice injected with control or AD fractions (Figure 4). However, the mean area covered by anti-phosphotau and anti-conformational tau immunoreactivity in these dystrophic neurites was significantly increased in 5XFAD mice injected with AD sarkosyl fraction compared to 5XFAD mice not injected or injected with control sarkosyl fraction (Figures 4J–L).

The increased tau immunoreactivity in plaque-associated dystrophic neurites was not detected by the anti-human tau antibody (Figures 1C–E) in 5XFAD-AD injected mice (Figures 1C–E) indicating that this increased tau immunoreactivity was not due to the accumulation of human tau (injected 3 months before) in the dystrophic neurites surrounding plaques. Human tau was detected by the anti-human tau antibody in mice expressing human mutant 1N4R tau (Figure 1F).

Only phosphorylated tau was present in plaque-associated dystrophic neurites as tau aggregates were not detected by Gallyas staining in these structures. NFT were not observed in 5XFAD mice injected with control or AD sarkosyl fractions whereas they were detected in neurons of mice expressing human mutant 1N4R tau (Figure 4, insets in Figures 4A1,B1, and C1).

### The Permeability of the Blood–Brain Barrier Is Increased in the Brain of 5XFAD Mice

Surprisingly, the effect of intravenous injection of AD PHF-tau proteins on neuroinflammation and on amyloid or tau pathology was only observed in 5XFAD brain and not in WT brain. Previous studies have shown that the blood–brain barrier has a higher permeability in the brains of 5XFAD mice, suggesting that PHF-tau proteins could enter more easily in 5XFAD brain than in WT brain (Kook et al., 2012; Batarseh et al., 2017). To confirm this observation in our mouse model, we analyzed the presence of mouse immunoglobulins around blood vessels in WT and in 5XFAD mice brains. We observed a diffuse immunoreactivity for mouse immunoglobulins around blood vessels in 5XFAD brain (arrows in Supplementary Figure S1B) but not in WT brain (Supplementary Figure S1A), indicating that the blood–brain barrier had a higher permeability in 5XFAD mice, leading to the extravasation of mouse immunoglobulins.

## Discussion

In this study, we show that a single intravenous injection of PHF-tau proteins induces a long-term neuroinflammation (as evidenced by increased astrogliosis and microgliosis) and increases amyloid-β and associated tau pathology in the brain of 5XFAD mice and is already apparent in the small cohort of mice analyzed in this study. In previous studies, WT (Rozenstein-Tsalkovich et al., 2013) and tau transgenic mice (Boimel et al., 2010; Rozenstein-Tsalkovich et al., 2013; Selenica et al., 2014) have been treated by subcutaneous or intraperitoneal injection of tau peptides or recombinant human tau proteins with adjuvants to study the effect of an active immunization protocol with repeated injections on tau pathology. An increased or a decreased neuroinflammation and the presence of anti-tau antibodies in their serum was observed in these immunized mice and a reduction of tau pathology was reported by this immunotherapy approach. After a single intravenous injection of native PHF-tau proteins from AD brains (in the absence of adjuvants), we observed an increased neuroinflammation in the dentate gyrus of 5XFAD mice in absence of anti-tau antibodies in their serum, suggesting that the observed neuroinflammation did not result from an immune reaction due to the generation of anti-tau antibodies. Neuroinflammation induced by lipopolysaccharide treatment stayed for a longer time in the brain of APP transgenic mice than in WT mice but neuroinflammation decreased after a few days in their brain (Herber et al., 2004). In our AD injected 5XFAD mice, neuroinflammation was still present 6 months after injection, suggesting that the increased neuroinflammation observed in these mice is not just due to a non-specific, transient inflammatory reaction induced by the injection of PHF-tau proteins. Moreover, this increased neuroinflammation was observed only in AD injected 5XFAD mice and not in the control injected 5XFAD mice, indicating that this increased neuroinflammation is due to PHF-tau protein injection. We hypothesize that the increased neuroinflammation was observed in AD injected 5XFAD mice and not in CTL 5XFAD mice or in AD injected WT mice because of a combined effect of the presence of PHF-tau proteins in the injected material and of the disruption of the blood–brain barrier in 5XFAD mice as we confirmed by demonstrating an extravasation of immunoglobulins in the latter mice, implying potentially a more easy access of intravenously injected PHF-tau proteins to the brain tissue in 5XFAD mice.

An interesting finding of this study is that intravenous injection of PHF-tau proteins from AD brains increased amyloid-β burden in 5XFAD mice. The role of neuroinflammation in the formation of amyloid plaques is not yet clearly understood as mouse models in which neuroinflammation was induced by activator of the immune system (such as lipopolysaccharides) showed unmodified, increased, or even reduced amyloid plaque formation (DiCarlo et al., 2001; Herber et al., 2004; Lee et al., 2008). The important neuroinflammation present in the dentate gyrus of 5XFAD mice injected with PHF-tau proteins could be responsible for the increased amyloid plaque burden in this area, since microglial cells have been suggested to be involved in the production of amyloid-β (Wegiel and Wisniewski, 1990; Baik et al., 2016; Spangenberg et al., 2019) and could thus be directly responsible for the increased amyloid burden observed in AD injected 5XFAD mice. Moreover, treatment of 5XFAD mice with fingolimod, an anti-inflammatory drug, induced a reduction of gliosis but also a reduction of the amyloid load, indicating that gliosis can affect the formation of amyloid (Aytan et al., 2016). However, we cannot discard the possibility that the increased amyloid burden is primarily responsible for the increased astrogliosis and microgliosis that we have observed in our AD injected 5XFAD mice since reactive astrocytes and microglial cells were mainly localized around amyloid deposits.

A previous study showed that extracellular human tau proteins added in the medium of primary cultures of neurons increases the production of amyloid-β (Bright et al., 2015). The latter result supports our *in vivo* observation and is compatible with the hypothesis that intravenously injected PHF-tau proteins entering into the brain through increased blood–brain barrier permeability could induce the increased amyloid burden observed in 5XFAD injected mice.

Interestingly, the accumulation of abnormally phosphorylated and conformationally modified tau was increased in dystrophic neurites surrounding amyloid deposits in PHF-tau injected 5XFAD mice compared to control injected 5XFAD mice. It can be speculated that the increased amyloid burden observed in AD injected 5XFAD mice led to the increased tau phosphorylation in plaque-associated dystrophic neurites. Numerous studies have shown that amyloid exacerbates tau pathology in tau transgenic mice expressing mutant APP/PS1 or by stereotaxic injection of amyloid in the brain of tau mice but, compared to the present study, different mechanisms of tau-amyloid interactions are involved in these models (Lewis et al., 2001; Oddo et al., 2003; Bolmont et al., 2007; Terwel et al., 2008; Héraud et al., 2014). Indeed, tau pathology developed spontaneously due to the genetically derived expression of human mutant tau and tau pathology was enhanced, but not induced, by amyloid-β in these previous models. A role for the increased neuroinflammation in the increase of tau pathology in plaque-associated dystrophic neurites is also possible, as suggested by other observations linking neuroinflammation and tau pathology (Asai et al., 2015; Maphis et al., 2015).

The development of a tau pathology in the brain has been described after intraperitoneal (Clavaguera et al., 2014) injection of brain homogenates or PHF-tau proteins from AD brain tissue. Besides this study, few information are available about extracerebral route of tau pathology transmission. Intracerebral injection of PHF in previous studies induced the formation of Gallyas and phosphotau positive fibrillary tau inclusions mainly as grains, neuropil threads, and glial inclusions (He et al., 2018; Vergara et al., 2019). In 5XFAD mice, an accumulation of phosphorylated tau in dystrophic neurites surrounding amyloid plaques was observed after injection of PHF-tau proteins from AD brains but without detection of tau aggregates in these dystrophic neurites (Vergara et al., 2019). In our study, the accumulation of phosphorylated tau in dystrophic neurites surrounding amyloid plaques and the absence of tau aggregates in these dystrophic neurites are in agreement with observations made in intracerebrally injected 5XFAD mice. However, a difference between intravenous and intracerebral injection of PHF-tau proteins is the absence of formation of tau inclusions in neurons or glial cells in our intravenous injection study. We cannot discard the hypothesis that tau inclusions could be present in our intravenous injected mice after longer incubation periods as tau aggregates developed spontaneously in APP/PS1 mice at older age as reported in a recent study (Metaxas et al., 2019). However, the absence of pericaryal tangles formation in 5XFAD mice after intravenous injection was not specifically due to the inoculation pathway as such pericaryal tangle was not observed in 5XFAD mice after intracerebral injection of pathological tau proteins from AD brains (Vergara et al., 2019).

## Conclusion

We show in this study that a single intravenous injection of PHF-tau proteins from AD brain was sufficient to induce a long-standing neuroinflammation in 5XFAD mice with documented effects on amyloid burden and associated tau pathology. Tau proteins have been detected in the blood using sensitive methods (Zetterberg et al., 2013; Chiu et al., 2017; Tatebe et al., 2017; Fossati et al., 2019; Lue et al., 2019), and our study suggests that blood products containing modified tau proteins might have a potential role in modulating the development of AD pathologies in the brain of elderly people in which the blood–brain barrier permeability is increased with aging. This study in an animal model thus provides important information about the relative AD risk of the extracerebral route that is medically relevant considering the wide use of blood products.

Several limitations of the present study will need however to be addressed in future studies, including confirmation of the results in larger cohorts to take into account variabilities with the administration route and additional assessment of the role of the blood–brain barrier. Additional direct comparative studies of the effects of intravenous and intracerebral inoculation experiments will be informative for assessing the relative efficiency of extra- and intracerebral routes in the development of tau pathology.

## Data Availability Statement

The raw data supporting the conclusions of this article will be made available by the authors, without undue reservation.

## Ethics Statement

This study on postmortem brain tissue was performed in compliance and following review and approval of the Ethical Committee of the Medical School of the Free University of Brussels. All studies on animals were performed in compliance and following approval of the Ethical committee for the care and use of laboratory animals of the Medical School of the Free University of Brussels.

## Author Contributions

SH and M-AF are both first authors since they contributed equally to the work. M-AF, VV, ZY, SM, and RD performed the experiments. M-AF, SH, and KL collected the samples and analyzed the data. J-PB, KA, and LP read and edited the final manuscript. KL designed and supervised the study. SH, M-AF, and KL wrote the manuscript. All authors read and approved the final manuscript.

## Conflict of Interest

The authors declare that the research was conducted in the absence of any commercial or financial relationships that could be construed as a potential conflict of interest.

## Supplementary Material

FIGURE S1 Extravasation of immunoglobulins was detected in 5XFAD mice brain. **(A,B)** Detection of mouse immunoglobulins in the brain of WT **(A)** and 5XFAD mice **(B)**. A diffuse immunostaining (arrows) is observed around blood vessels (star) in 5XFAD mice whereas no immunoglobulin extravasation was detected in WT mice around blood vessel (star). Scale bar: 50 μm.
