# Supplementary figures and images for: Intravenous Injection of PHF-Tau Proteins From Alzheimer Brain Exacerbates Neuroinflammation, Amyloid Beta, and Tau Pathologies in 5XFAD Transgenic Mice
Source: Front Mol Neurosci. 2020 Jul 14;13:106. doi: 10.3389/fnmol.2020.00106 (PMC7381181; doi:10.3389/fnmol.2020.00106)

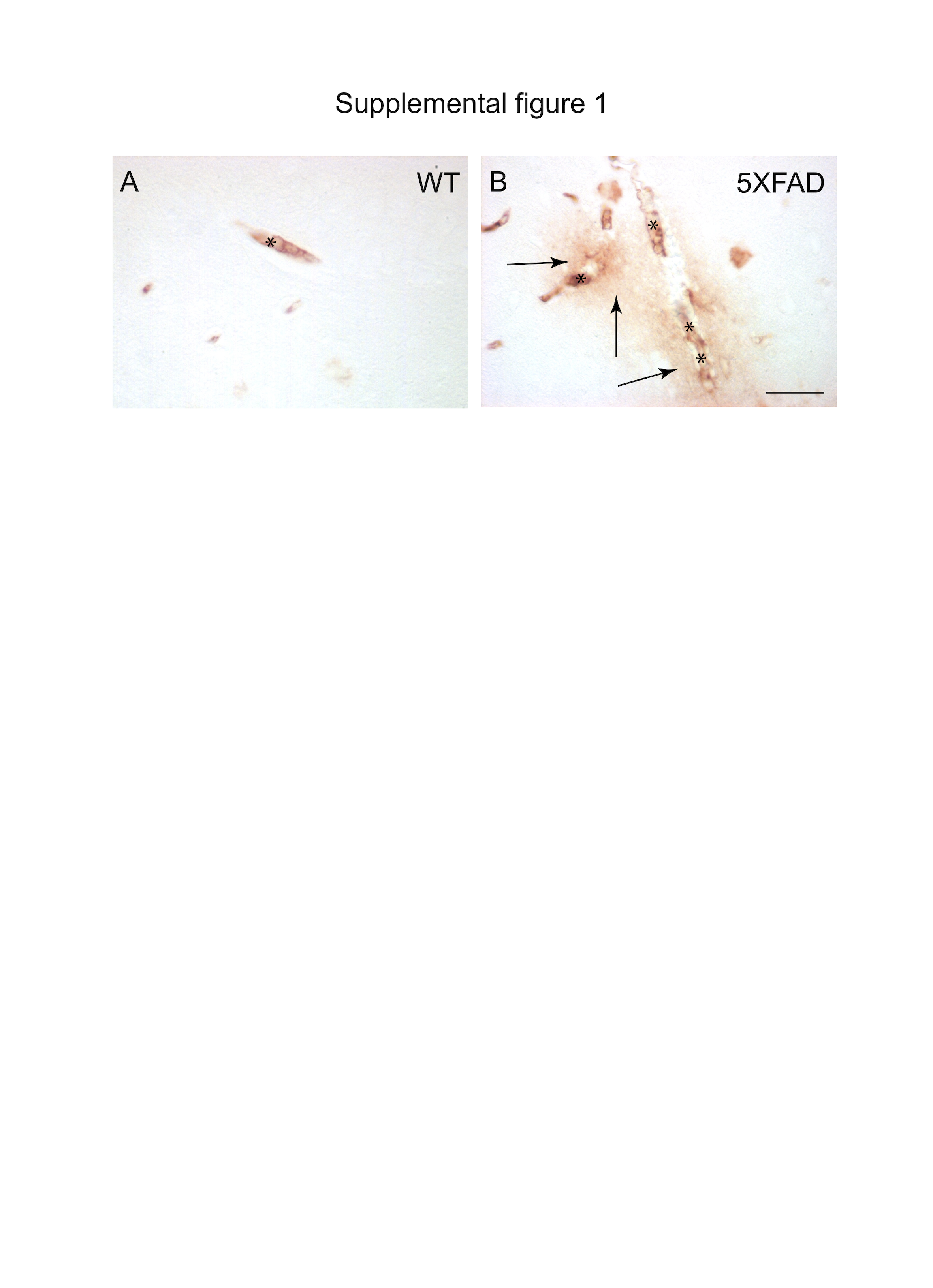

Supplement: FIGURE S1 — Extravasation of immunoglobulins was detected in 5XFAD mice brain. (A,B) Detection of mouse immunoglobulins in the brain of WT (A) and 5XFAD mice (B). A diffuse immunostaining (arrows) is observed around blood vessels (star) in 5XFAD mice whereas no immunoglobulin extravasation was detected in WT mice around blood vessel (star). Scale bar: 50 μm. [file Image_1.TIF]
